# Right ventricular involvement in acute myocardial infarction. Risk stratification by visualization of wall motion, edema and delayed enhancement cardiovascular magnetic resonance

**DOI:** 10.1186/1532-429X-13-S1-P116

**Published:** 2011-02-02

**Authors:** Matthias Grothoff, Christian Elpert, Janine Hoffmann, Suzanne de Waha, Ingo Eitel, Holger Thiele, Matthias Gutberlet

**Affiliations:** 1 University of Leipzig - Heart Center, Leipzig, Germany

## Introduction

Patients with RVI complicating AMI suffer from increased morbidity and mortality, but it is unclear which patients are in risk of developing RVI. Cardiovascular magnetic resonance (CMR) can identify patients with RVI and might add important information for risk stratification, prognosis and treatment.

## Purpose

We aimed to determine the predictors of right ventricular involvement (RVI) assessed by wall motion abnormalities, edema, myocardial salvage and delayed enhancement in acute reperfused myocardial infarction (AMI) and the prognostic significance of RVI.

## Methods

We studied 431 patients 1-4 days after primary angioplasty for ST-elevation AMI. T2-weighted and delayed enhancement (DE) CMR was used for visualizing edema and scar to calculate myocardial salvage (MS). Cine-imaging was used for wall motion analysis. Patients with RVI were compared to matched patients with isolated left ventricular (LV) infarction. The primary endpoint was the occurrence of a major adverse cardiac event (MACE): a composite of death, reinfarction and congestive heart failure.

## Results

RVI with localized wall motion impairment and edema was found in 69 patients, of which 41 showed myocardial necrosis in DE imaging. In multivariate linear regression analysis predictors of RVI were a low TIMI-flow grade before angioplasty (OR 3.93, 95%CI 1.16-13.29, p=0.03), a high RV myocardial mass (OR 1.1, 95%CI 1.01-1.20, p=0.03) and a low ST segment resolution (OR 2.32, 95%CI 1.06-5.05, p=0.03). In Cox regression the strongest predictor of MACE was RVI (HR 3.66, 95%CI 1.99-5.66, p<0.001).

## Conclusions

RVI detected by CMR is a strong and independent predictor of clinical outcome after acute reperfused AMI.

**Figure 1:**
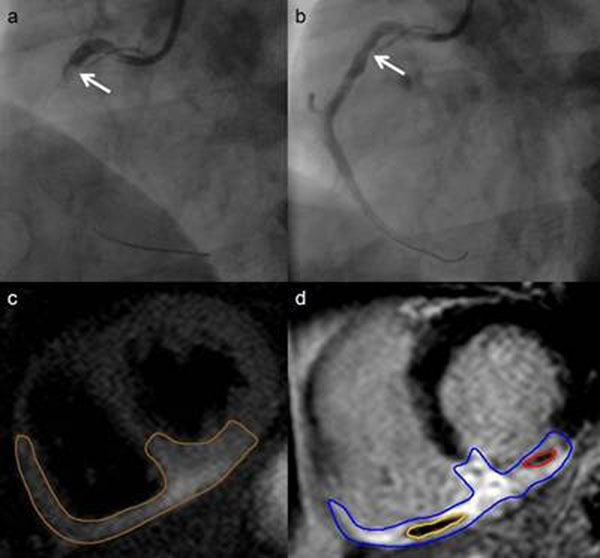
Coronary angiography and CMR of a severe inferior infarction: **a** proximal occlusion of the RCA, after PCI **b** with post TIMI-flow II. Area at risk (brown contour) extends from the LV inferior wall to the interventricular septum and the RV inferior and free wall in **c**. In **d** MO of the RV (yellow contour) and the LV ( red contour) is shown.

